# The golden ratio in baseball: the influence of historical eras on winning percentages in major league baseball

**DOI:** 10.3389/fspor.2023.1273327

**Published:** 2023-11-14

**Authors:** John Cairney, Stephen Townsend, Denver M. Y. Brown, Jeffrey D. Graham, Veronique Richard, Matthew Y. W. Kwan

**Affiliations:** ^1^ School of Human Movement and Nutrition Sciences, The University of Queensland, Brisbane, QLD, Australia; ^2^ Health and Well-Being Centre for Research Innovation, The University of Queensland, Brisbane, QLD, Australia; ^3^ College for Health Community and Policy, University of Texas at San Antonio, San Antonio, TX, United States; ^4^ Department of Child and Youth Studies, Brock University, St Catharines, ON, Canada

**Keywords:** baseball, golden section, audience, fans, statistics, history, championship

## Abstract

**Introduction:**

The golden section or golden ratio (61.8% or 0.618) is a mathematical phenomenon that appears in art, literature, music and nature with such ubiquity that it is thought to be a fundamental principle of aesthetic organisation. The golden ratio also manifests in sport, particularly as the proportion of wins to losses required to win a Major League Baseball championship. This study extends early work on the golden ratio in baseball by incorporating more than three decades of additional data.

**Methods:**

This study involved a historically contextualized examination of how winning percentages have changed across the seven historical eras of modern baseball, including analyses of the relative contribution of offensive and defensive statistics to championship winning teams. Data was extracted from Baseball Reference and included statistics for 398 championship winning teams from both the American and National Leagues between 1901 and 2019. Pearson correlation coefficients were computed for winning percentage with indicators of offensive and defensive performance during each era. Main and interaction effects of Era and League on winning percentage were examined using factorial ANOVA, with follow-up analyses examining whether the golden ratio was included in each factor's 95% confidence interval.

**Results:**

Our findings suggest that winning percentages for championship teams were closest to the golden ratio during eras where the relative contribution of offense and defense was most closely balanced: the Integration Era (1942–1960) and the Expansion Era (1961–1976).

**Discussion:**

Previous scholarship theorizes that the golden ratio represents an aesthetic ideal or a Gestalt archetype. If this aesthetic theory is applied to sporting competition, these results suggest that baseball may be most aesthetically appealing to fans when offense and defense is balanced in such a way as to ensure that championship teams win 61.8% of their games.

## Introduction

The golden section, or golden ratio as it referred to in this paper, is the relationship between two elements (a and b), defined by the following formula: a/b = b(a + b), where a + b = 1. The relationship of the smaller (a) to the larger (b) is the same as the relationship of the larger to the sum of both elements (a + b). In its simplest terms, the golden ratio hypothesis proposes that when an object or quantity is divided into two *unequal* parts, the larger element represents 61.8% of the whole. This proportion appears in nature and art with such ubiquity that it has been considered for millennia to be a fundamental, although mysterious, principle of aesthetic organisation. For example, since the time of the Ancient Greeks, the golden ratio has been widely considered the most aesthetically pleasing way to divide a line and it is from this context that the term “golden *section*” most likely originated.

The phenomenon has since been observed in non-geometric contexts, including numerous works of art, from the paintings of Leonardo Da Vinci to the musical compositions of Debussy (1). In these contexts, the term golden *ratio* is arguably more appropriate than golden section. In this study, we employ the term golden ratio because it more appropriately describes the mathematical and historical relationships we are analysing; win to loss ratios for championship-winning Major League Baseball teams. We extend our analysis to examine how the changing historical relationship between offensive and defensive production creates conditions which bring about the golden ratio. The implications of golden ratio in baseball are discussed in the context of fan engagement and the shifting popularity of baseball across its historical eras.

Beyond the simple fact it appears pleasing to the eye, the golden ratio also turns out to have psychological manifestations, perhaps for the same underlying reasons. Researchers have suggested the golden ratio appears to influence cognition under specific conditions. Benjafield and Adams-Webber (2) for example, formulated the Golden Section (Ratio) Hypothesis, which predicts that when individuals are compelled to divide something into unequal parts, they tend to do so using the golden ratio. Numerous experimental studies have provided support to this hypothesis (3). The tendency to employ the ratio when compelled to make unequal decisions suggests a possible psychological explanation for why it is manifested in art and in experimental psychological studies: We are drawn to the asymmetry, if not the beauty, that this ratio produces and tend to organize events and experiences according to its proportions.

If the golden ratio is indeed so ubiquitous, we would expect it might be present in multiple human endeavours. Sport may be one example. Benjafield (4) hypothesized that the deliberately unequal conditions of sporting contests may create manifestations of the golden ratio. In particular, Benjafield argued that sporting contests create winners and losers in unequal proportions, with the former being much more valued than the latter. He further hypothesized that in league conditions, where competitors accumulate points for defeating a sequence of opponents across a “season”, the win to loss ratio required to win a championship might correspond with the golden ratio. To test this theory, he examined Major League Baseball statistics between 1901 and 1985, and revealed that the average win to loss ratio for championship-winning teams over this time period corresponded exactly with .618.

Baseball is a particularly interesting context in which to further investigate this phenomenon. The regimented nature of baseball gameplay produces hundreds of discrete units of play in every match, making it relatively easy to quantify the performance of individual players and teams. As a result, nuanced aspects of the game have been quantified and recorded for more than a century, and this extensive record keeping allows for detailed examination of this phenomena at arguably the highest level of baseball competition. Furthermore, the basic rules of baseball have remained consistent since the turn of the 20th century allowing researchers to reliably identify and analyse performance trends across the modern era (5). The static nature of baseball's basic rules is such that structural or collateral factors have historically had a significant influence on gameplay, so significant in some cases that they have produced distinct “eras” in the game. For example, changes to the composition of the ball gave rise to the Live Ball Era (1920–1941), the recruitment of players from the Negro Leagues initiated the Integration Era (1942–1960), and the proliferation of ergogenic aids between 1994 and 2005 produced the home-run bonanza known as the Steroid Era (6). Woltring et al. (6) argue that baseball's eras are marked not only by historical events but can also be identified by statistically significant changes to on-field performance.

Why would wins in baseball (or any sport) conform to the golden ratio? Benjafield (4) hypothesized a social mechanism whereby winning and losing would be influenced by the presence of fans. Having a winning sport team is also highly valued and a point of pride for individuals who live in the communities the teams represent. Fan expectations have been shown to influence winning and losing (7). In particular, teams competing at home, on average, win more often than visiting teams (8, 9) Supportive home audiences are one reason this is so. Following this, Benjafield (4) argued that the golden ratio might be the “optimal” number of wins required before a team is considered to be a champion and thus valued by its supporters: the percentage of wins should, on average, approximate.618 or 61.8%, if the golden ratio is indeed an influencing factor for the perception of champions. This theory may apply to other sports. For example, Klugman (10) suggests that when an Australian Rules Football club wins a premiership, its fans develop an irrational passion for their team that is akin to romantic love. However, this irrational and inexplicable love can only develop if the team has overcome trials during the season. In other words, the team must win but cannot completely dominate. The golden ratio of 61.8% may be the ideal winning proportion for fans to “love” their team.

However, the influence of fans on game play may be constrained by the physical dimensions of the playing surface. Benjafield (4) argued that the physical proximity of the fans to the playing field would have a different effect on the golden ratio (percentage of wins), based on what has been termed the audience closeness effect (4, 11). Benjafield (4) supported this theory by comparing win ratios in baseball to those of ice hockey, which has markedly different physical conditions governing fan-player interaction. In baseball parks, the fans are further away from on-field play (on average) but in ice hockey, fans are immediately adjacent to the ice, separated only by boards and a glass surface. Benjafield (4) predicted that for baseball, the proximity of fans would be less important and so wins would follow the golden ratio for all games, regardless of whether they are played at home or away. In other words, a championship team would win, on average, 61.8% of the games played for a season. For ice hockey, where fans are close, he predicted wins at home (not away) would follow the golden ratio. Using archival data for both sports, Benjafield (4) indeed found that the overall ratio wins in baseball was 61.8%, whereas only home games in ice hockey conformed to this pattern.

But why would wins under these conditions follow the golden ratio? Here Benjafield (4) offers a hypothesis derived from Jungian and Gestalt psychology. In the first instance, the ubiquity of the golden ratio suggests it may be archetypal, a postulation similarly made by Lutz (12). As observed in the studies previously described, the golden ratio might represent an archetype that governs the organization of perception and experience related to unequal allocations. However, Benjafield (4) argues that the archetype more closely follows Gestalt laws of organization (e.g., law of symmetry) in that the golden ratio as an organizing principle is influenced by context: “Just as the Gestalt laws are not rigid mechanisms which are insensitive to circumstances, so the Golden Section [ratio] would inform different aspects of experience depending on the particular context” (p. 112). In other words, the golden ratio is a “universally available” means of “organizing experience”, so ubiquitous it can be employed without conscious reflection. It is experienced by individuals as “natural” and becomes a “collective norm”, but only under circumstances that allow for it. In the context of sport, Benjafield argues that the golden ratio manifests only if a team's supporters are consistently close enough to positively influence their gameplay.

Benjafield's fan-proximity theory is statistically and psychologically compelling but it is unlikely to offer a full explanation. It also presents a somewhat cyclical dilemma, in that it is not clear whether the influence of vociferous supporters enables championship teams to win 61.8% of the time, or whether teams that win 61.8% of the time are more likely to attract enthusiastic fans. It is possible that over a period of 120 years, the success of championship-winning baseball teams and the relative enthusiasm of their fans has interplayed in a complex feedback loop that finds its level at a 61.8% win ratio. There are, however, other related factors that should be considered. As outlined previously, the history of baseball can be characterized by distinct chronological eras. The boundaries of these eras are marked by paradigmatic historical events, and later statistical analysis has also revealed that the balance between offense (hitting) and defense (pitching) changes from era to era (6). Next, we thus consider these statistical shifts in the context of the golden ratio and its relation to baseball fandom. In doing so, we query whether gameplay in one or more of these eras achieved an “optimal” balance between offense and defense which was more appealing to fans.

Baseball offers a rich case study to examine the potential relationship between winning percentage, the golden ratio, and historical gameplay conditions. The relative balance between the game's oppositional foundations—offense (hitting) and defense (pitching)—has frequently changed over time, allowing us to chart the chronological relationship between winning percentage and the balance of offense and defense. Historians and statisticians have previously identified distinct periods or “eras” when one of these elements tended to dominate. For example, the so-called Dead Ball era represents a period where pitching very much dominated in the game, in part owing to the rules of the game of that time but also a claim that the way in which the ball was constructed itself made it less “lively” and therefore more difficult to hit for distance. The end of the Dead Ball era corresponds with the emergence of Babe Ruth and power hitting, the so-called Live Ball era, where we see a shift from pitching dominance to offense, characterized by hitting for extra bases and home runs. Much later, the widespread use of steroids between 1995 and 2005 (the Steroid era) created competitive imbalances between those players that used ergogenic aids and those who did not, once again changing features (i.e., power hitting) which affected how games were won (13).

Recently, Woltring and colleagues (6) provided an empirical validation of these historical eras by examining the relative contribution of offense and defense to wins, building from Bill James' Pythagorean Expectation statistic (14). James argued that winning teams should score more runs than they allow across a season, and expressed this has a ratio as follows: 1 / 1 + (runs allowed/runs scored)^2^. While it is possible to use runs scored and runs allowed to predict winning, an alternative approach is to consider what influences run production and runs scored at the team level. Hakes & Sauer (15) argue that two statistics above all else—slugging (SLG) and on-base percentage (OBP)—account for most of the variance in winning percentage. Building on this observation, Woltring and colleagues (6) employed on-base plus slugging (OPS), which combines the hitting team's On Base Percentage (OBP) and Slugging Percentage (SLG) into a single statistic, providing a robust measure of offense (how runs are scored). Indeed, Hakes & Sauer (15) report a high correlation between these two indicators and run production. For defense (how runs are allowed), the researchers used the equivalent pitching statistic measures how many times the pitching side allowed opposition hitters to get on-base (OBPa), and how often they allowed hits for power (SLGa).

Woltring and colleagues found a statistically significant interaction between historical era and OPS, and era and OPSa (including OBPa for 1901 to 1949) when predicting winning percentage. Overall, when comparing the relative contributions of offense and defense to winning percentage, the results for five of the seven eras aligned with historical interpretations of the factors most associated with successful teams in each respective era. For example, as noted above, the Dead Ball era is associated with pitching (defense) dominance. Consistent with this, Woltring et al. (6) found OBPa to be more strongly correlated with win percentage than OPS, meaning that teams which allowed fewer opposition players to get on base were more likely to be successful than teams with good hitting and on-base percentages. In simple terms, the perception that defense was more important than offense during the “dead ball era” is confirmed by statistics. Moreover, the absolute difference between the correlations representing offense and defense and win percentage were largest in this era.

Similar support (results consistent with historical interpretations) was found for the integration era, the expansion era, the free agency era, and the post-steroid era. The exceptions were for the live ball era and the steroid era. For the former, the historical account for that era suggests that hitting drove wins, but in fact, pitching contributed more to wins than hitting. Similarly, the steroid era also shows that despite the commonly held belief that hitting was responsible for wins during this period, pitching was more strongly correlated to wins in this era.

The validation of winning percentage attributable to the relative contributions of pitching versus hitting over time by Woltring and colleagues (6) provides an important empirical basis for examining the expression of the golden ratio over the history of the modern game. Because Woltring et al. (6) have established there are periods where the relative differences between offense and defense to winning are smaller (e.g., during the expansion era) and periods where the relative differences are larger (e.g., during the deadball era), we can designate periods where the relative difference between offense and defense as periods of significant deviation from ideal balance (i.e., one is dominant over the other). Aesthetically, much like the golden ratio creates an ideal proportion, when the two opposing forces of a sport are in balance, when neither defense nor offense dominates a championship teams' success, the condition is ideal and therefore, championship teams will win at a rate consistent with the golden ratio. In other words, a win rate consistent with the golden ratio will reflect conditions of ideal balance in the foundational oppositional elements of the game.

This of course only holds true if, as has been demonstrated elsewhere, the golden ratio in fact represents a universal ideal (4). We describe this as the aesthetic of competitive balance hypothesis: *when the relative contribution of offense and defense to winning percentage is balanced, championship teams will tend to win games at a proportion consistent with the golden ratio*.

To test this hypothesis, it must first be established that the winning percentage of championship teams will only be equal to the golden ratio, when the balance between offense and defense is ideal. During periods of imbalance between offense and defense, when one or the other makes a greater contribution to winning championships, the win percentage for championship teams will fall outside the value for the golden ratio. To date, existing studies have not considered whether the golden ratio deviates over time, through clearly defined historical periods such as the eras noted here.

Following Benjafield (2–4) and using the validation results provided by Woltring et al. (6) to identify distinct historical eras of the game that consider the relative contribution (and therefore balance) between offense and defense, this study determined whether win percentage among championship teams deviated from the golden ratio during eras where there is greater imbalance between offense and defense. We hypothesized that eras in which there is greater imbalance between offense and defense will have championship teams with greater winning percentage deviations from the golden ratio of .618.

## Methods

### Sources of data and data extraction

Data on winning percentage among championship teams were extracted from Baseball Reference (baseballreference.com). The dataset included a complete record of wins and losses (among other statistics) for the American and National Leagues from 1871 to 2021. For the purposes of this analysis, we confined our analysis to between 1901 and 2019. The rationale for beginning in 1901 is that it reflects the year that most baseball scholars consider the start of modern professional baseball. This was the year that witnessed the establishment of the dual-championship structure that continues to this day (16, 17). The 2019 season was selected as the final season for analyses, as the seasons following had significant and ongoing disruptions caused by the COVID-19 pandemic. With our final dataset, our sample included seven eras: the Dead Ball Era (1901–1919), the Live Ball Era (1920–1941), the Integration Era (1942–1960), The Expansion Era (1961–1976), the Free Agency Era (1977–1993), the Steroid Era (1994–2005), and the Post-Steroid Era (2006–2019).

Baseball Reference has been shown to be highly reliable. In a previous study, the accuracy of the site's data was examined by comparing a 1% sample of player-level data, extracted randomly from the inception of the data to 2018 (*n* = 9,775), to the official records in MLB.com (Brown et al., 2019). The Baseball Reference data was shown to be 94.5% accurate. It should be noted that in this analysis, the comparison was based on player-level performance statistics (e.g., OPS, OPS+), which are more difficult to collect and calculate (based on a number of performance indicators); and therefore more prone to errors, than wins and losses, which are routinely collected and included in box scores.

It should also be noted that when comparing over time, the structure of MLB changed slightly and so have the number of championship teams. From 1901 to 1968, there were only two championship teams, one from each League. From 1969 to 1993, the number of championship teams increased to four as both Leagues were divided into East and West divisions. From 1994 to the present a Central division was added, bringing the total number of championship teams to six. The total number of championships in the analysis was 398, 199 in each league.

### Data analysis

First, to determine the relative contribution of offense and defense during each respective era, absolute differences between the Pearson correlation coefficients for OPSa-winning percentage and OPS-winning percentage (consistent with methods established by Woltring et al. (6) were computed. OPSa data were not available prior to 1950 so for the period prior from 1901 to 1949, the authors relied instead upon OBPa only. In these analyses, OPS represents the offensive (hitting) and OPSa/OBPa represents defensive (pitching) contributions to winning percentage. Next, descriptive statistics for win percentage for all championship teams from American and National Leagues were computed and the difference in average winning percentage between leagues was compared using an unequal variances (Welch's) t-test. A 7 × 2 (i.e., Era by League) factorial ANOVA was then computed to examine the main effects of both Era and League and the Era x League interaction for winning percentage. Finally, the 95% confidence intervals for each era by league were examined to see whether they included the golden ratio value (.618).

## Results

The results of the relative contribution of offense and defense are summarized in Table 1 [these results come directly from Wolting et al. (6) except for absolute differences which have been calculated from the results provided]. The negative correlation for OPSa/OBPa simply reflects the association of the statistics (in this case, OPS of the opposing team) to winning. It is the absolute value that should be compared across statistics. Taking the Dead Ball era as an example, the correlation for OPSa/OBPa (-.619) has a stronger relationship to winning percentage than OPS (.540). Further, 38% of the variability in winning percentage can be accounted for by pitching performance (defense) compared to 29% of the variability explained by offense. From this, three trends are apparent. First, among the seven eras examined, three showed a relative advantage of defense over offense in relation to winning. Second, the largest differences between offense and defense occurred during the Dead Ball and Steroid eras. In both eras, pitching had a stronger correlation with winning than offense. Third, the smallest difference between offense and pitching to winning occurred during the Expansion era, where the relative impact of both on winning was about the same. The relative difference between offense and pitching in terms of winning was also relatively small in both the Live Ball and Integration Eras.

**Table 1:** Relative contribution of OPS and OPSa/OBPa to winning percentage across different eras.

Era	OPS	OPSa/OBPa	Absolute difference	Relative contribution of offence to defence
Dead ball (1901–1919)	.540***	−.619***	0.079	D > O
Live ball (1920–1941)	.603***	−.615 ***	0.012	D > O
Integration (1942–1960)	.643***	−.616 ***	0.027	O > D
Expansion (1961–1976)	.553***	−.548 ***	0.005	O = D
Free agency (1977–1993)	.554***	−.507 ***	0.047	O > D
Steroid (1994–2005)	.567***	−.644 ***	0.077	D > O
Post-steroid (2006–present)	.522***	−.564 ***	0.042	D > O

***Indicates a *p*-value of < .001. High statistical reliability.

The win percentage for all championship teams from 1901 to 2019, was.606 with a 95% CI of 0.602 to 0.610. For the National League specifically during the same period, winning percentage was 0.603 with a 95% CI of 0.597 to.609. The winning percentage in the American League was 0.608 with a 95% CI of 0.601 to 0.614. The difference in average winning percentage between leagues was not statistically significant [*t*(390) = −1.223, *p* = .222].

**Figure 1:**
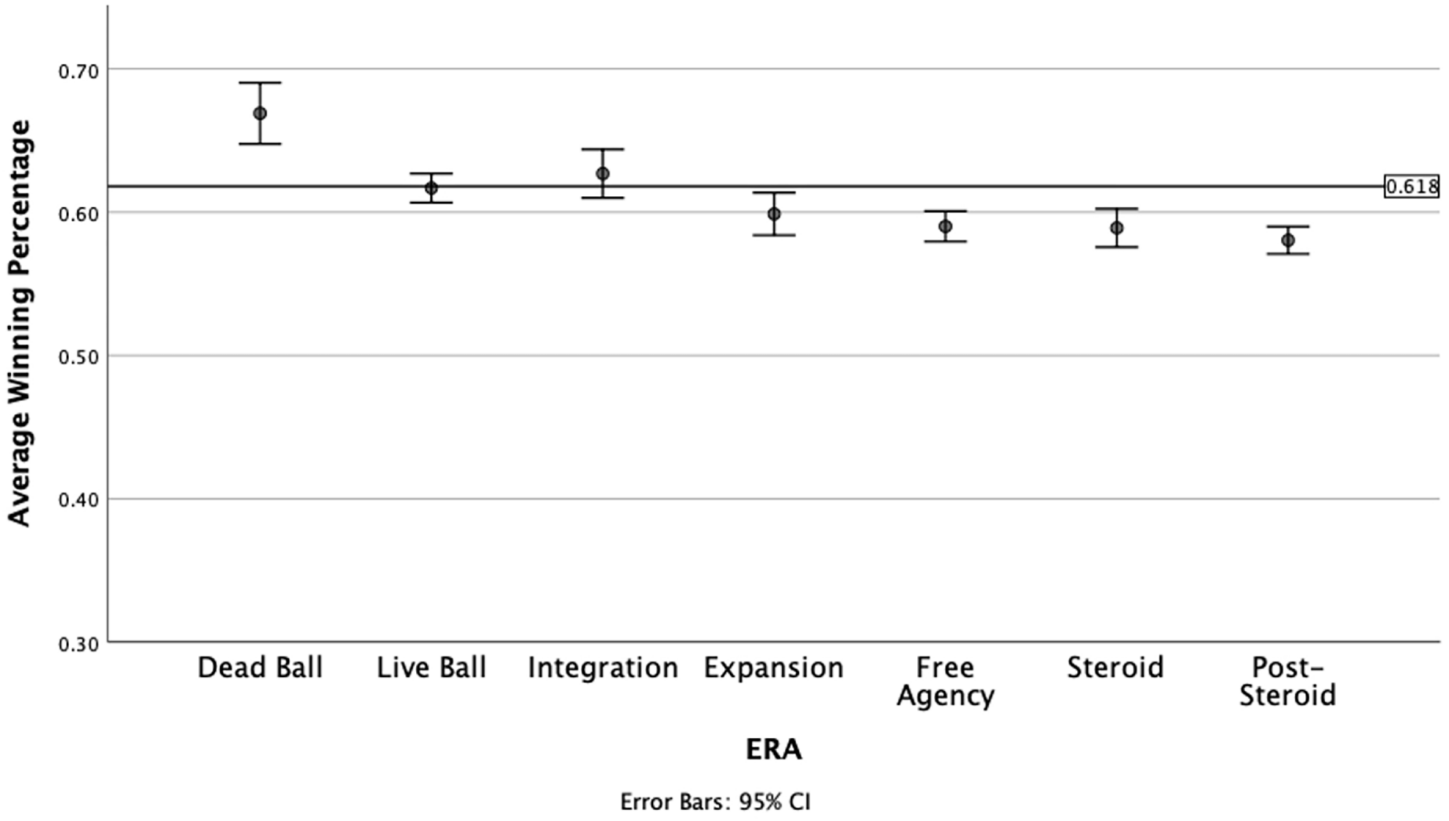
Winning percentage for national league champions by Era, 1901 to 2019.

**Figure 2:**
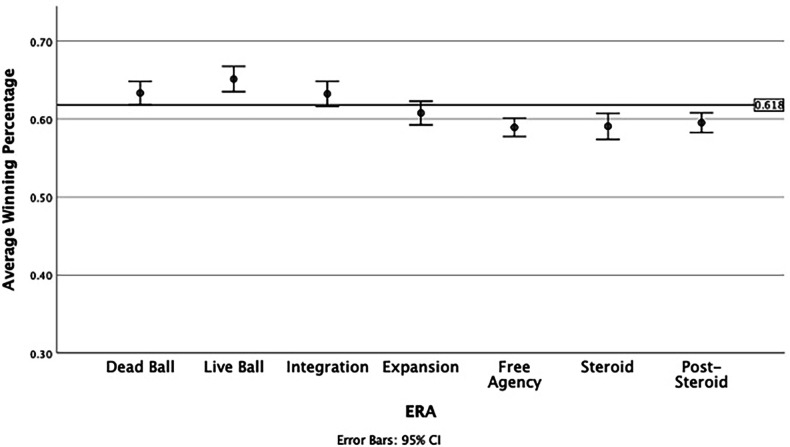
Winning percentage by Era in the American League, 1901 to 2019.

Next, we examined the main effects of both Era and League, and the interaction effect between these variables using a 7 by 2 Factorial ANOVA. All effects were significant at the *p* < .05 level except for the main effect of League [*F*(1,378) = 1.121, *p* = .290]. The main effect for Era was significant [*F*(6,378) = 24.567, *p* < .001], indicating winning percentage varied across different eras. Importantly, the interaction effect for Era by League was significant, indicating the relationship between era and winning percentage differed by league [*F*(6,378) = 3.502, *p* < .001]. To aid our interpretation, we produced two graphs showing the change in winning percentage across eras for both leagues separated. These are shown in Figures 1, 2 below.

In the National League, only two eras include the golden ratio within the 95% CI for winning percentage—the Live Ball and Integration eras. The Expansion era was close to the golden ratio, with the upper bound of the confidence interval just below the value of.618. While the Dead Ball era winning percentage was much higher than the golden ratio, the more recent Free Agency, Steroid and Post-Steroid eras had average winning percentages that fell well below the golden ratio value. According to the results provided by Woltring et al. [6] summarized in Table 1, the Integration era is a period where the absolute difference between OPS and OPSa is smallest (.005) and therefore, the relative contribution of offense and defense is equal. The Live Ball era shows a slight advantage of defense, but the absolute difference is the second smallest next to the Expansion era.

A slightly different picture emerges when examining the American League over the same time scale. Three eras—Dead Ball, Integration and Expansion—all had winning percentages and CI intervals that included the golden ratio value. The greatest deviation from the golden ratio value was observed for the Live Ball era. Similar to the National League, however, the more recent Free Agency to Post-Steroid eras had average winning percentages that were below the golden ratio value. When compared against Table 1 results, no consistent pattern to relative balance between defense and offense can be discerned. The Dead Ball era for example shows a clear effect of pitching being more important for wins than hitting. The Integration era shows the opposite. Only the Expansion era is consistent between leagues showing a balance between offense and defense, and a winning percentage that includes the golden ratio.

## Discussion

Overall, we extended Benjafield's [4] work by showing that the average winning percentage for a championship team has declined since 1985, but also since the inception of modern professional baseball in 1901 with declines observed across both Leagues. While the pattern varies somewhat between Leagues, overall, there has been a decline in winning percentage for championship teams with averages being above or close to the golden ratio in the early to mid-half of the 20th century and average values declining below the golden ratio and levelling-off in the latter half of the century and into the 21st century. From 1901 to 1985, the total winning percentage in both Leagues was 0.621 (95% CI: 0.616, 0.626), meaning the golden ratio falls within its confidence interval [4]. In fact, the winning percentage for National League championship teams was exactly 0.618 (95% CI: 0.610, 0.626) and slightly higher for the American League at 0.621 (95% CI: 0.616, 0.626) [4]. Including the period since 1985 to 2019, winning percentage overall has dropped to 0.606, and the confidence intervals for both Leagues do not include the golden ratio of 0.618. Overall, winning percentage has declined over time in both Leagues, and since the era of free agency, it has remained below the 61.8% criterion.

Based on the analysis conducted by Woltring et al. [6], there is only one era—the Expansion era—with parity in the relative contribution of offense and defense to winning. In every other era, either defense or offense had a stronger influence on team success. We postulated that if balance between offense and defense represented an ideal balance (the aesthetic of competitive balance hypothesis), then those eras would be more likely to produce championship teams with winning percentages equal to the golden ratio. Our hypothesis was only partially supported; and somewhat varied by league. In fact, only three eras produced championship winning percentages approximating the golden ratio: the Integration era (in both leagues), the Live Ball era in the National League, and the Expansion era in the American League. The Deadball era was also very close in the American League. Overall, we must conclude that balance between offense and defense then, may be of only limited importance in terms of achieving the golden ratio.

It is perhaps noteworthy that the Integration era is the only period where average winning percentage for championship teams approximated the golden ratio in both leagues. Beginning with the entry of Jackie Robinson into the Majors, the Integration period saw a rapid increase in the number of African American baseball players, including many stars from the Negro League. The Integration era was also a time where offense dominated, suggesting that the newly included African American players made their most significant contributions either at the plate or on the base-paths. Jackie Robinson, for example, was certainly known for his aggressive base running [18]. The players who immediately followed him were also recognized for their offensive prowess. The hitting of Larry Doby, for example, was largely responsible for the Cleveland Indians' victory in 1948 World Series [19]. Of course, Doby's teammate in that World Series win and former star of the Negro Leagues—Satchel Paige—was a pitcher. However, by the time Paige was signed by the Indians he was (at least) 42 years old and his once-substantial defensive performance was in decline [20].

It is important to note that the commonly held view of the Integration Era as an offensively dominated period was (and continues to be) influenced by racist assumptions about the heightened physicality of African American athletes. The findings of this study do not support perceptions of mid-century African American players as disproportionately offensively oriented, but these findings may indicate the outsized influence of exceptional players like Robinson and Doby, whose offensive prowess *is* reflected in their career statistics. It is also intriguing to hypothesize the relationship between player diversity and the golden ratio. As the game became more open to all players from the Negro Leagues and eventually to Latino players, it is possible that a greater diversity of experiences, strategies, temperaments, and skillsets produced winning ratios which closely approximated the golden ratio. As the game became more exposed to a greater diversity of players, winning followed the golden ratio.

Conversely, the periods in both Leagues where winning percentage fell below the golden ratio, were eras marked by significant structural changes to labour rights, the rampant use of performance enhancing drugs, and a significant increase in offense driven by power hitting. Free agency marked the end of the controversial reserve clause, which bound players to teams for the entire duration of their careers [21]. This resulted, in theory in a competitive advantage for those teams with the economic resources to recruit high quality free agents [22]. Indeed, there is some evidence to support that following removal of the clause, team performance and revenue increased with player movements [23]. Similarly, the Steroid and Post-Steroid eras also represented significantly disruptive periods. One fueled by performance enhancing drugs, the other marked by increasing offensive production, possibly related to changes in the composition of the ball itself. Further changes to offensive production are likely to be felt in coming years, as MLB technicians fine-tune equipment tolerances. As shown by an MLB scientific commission revealing that the 2019 home-run boon was caused by a manufacturing inconsistency in Rawlings baseballs, seemingly minor alterations can cause significant gameplay changes across the course of a season [24].

Of course, as James observed [14], the designation of eras in baseball can be based on any number of different factors, depending on the perspective and interest of the historian. The designation of a baseball era is not always determined by wins and losses *per se*. Some eras are characterized by historical events which caused paradigm shifts in the administration or management of the game and its players. For example, the so-called Expansion era corresponds to a time when the leagues expanded, adding teams in California for example, thereby increasing the geographic reach of professional baseball across the country. The Integration era saw Jackie Robinson break the colour barrier in Major League Baseball, paving the way for other teams to sign African American players. While these eras are defined by distinctly different events or circumstances (as almost all eras are), we could nevertheless reasonably expect such historically specific conditions to affect how games are won. The Expansion era for example introduced a new variable—travel. Indeed, research has shown travel across different time zones can disrupt sleep, leading to fatigue resulting in poorer performance [25]. Similarly, the influx of talented African American (and eventually Hispanic) players during the Integration era, led to increased competitiveness between teams, raising overall talent levels in the game [26]. It would seem therefore that structural and social changes to baseball administration and management translated to changes in gameplay, resulting in a periodic rebalancing of the ways that games are won (or lost).

To understand implications of these findings, we are required to accept that the golden ratio represents an archetype of cognitive and aesthetic preference. For example, this analysis has potential implications for sporting organisations (particularly professional baseball organisations) who wish to understand under which conditions their leagues are most appealing to fans. If we take Benjafield's [4] theory about the relationship between fan influence and competitive aesthetics, we could argue that baseball was most appealing to fans during the “eras” where the balance between offense and defense produced win ratios of approximately 61.8%. Our analysis suggests that these eras, and the structural changes upon which they are predicated, influenced win to loss ratios in complex ways, causing them to periodically deviate from the golden ratio. If Major League Baseball wants to increase its spectator appeal, an important consideration given its declining audience, it may consider implementing structural and gameplay conditions which ensure that championship teams win 61.8% of their games during a season. We have suggested some factors (particularly related to the measures of offensive and defensive production) which may have historically caused winning percentages to deviate from this number. However, further analysis is needed to answer the questions prompted by our analysis.

We could alternatively argue that the decline in winning percentage for championship teams over time, from an average close to 0.618 to one inching closer to 0.600 represents nothing more than greater parity in the league. Over time, the absolute differences (variability) in skill and performance have diminished, resulting in a more homogenous pool of highly talented players, increasing the level of competitiveness across all teams. This makes it much harder to dominate and championship winning percentages are consequently less lopsided. To many administrators and fans, lower win percentages may instinctively seem to be desirable condition because these numbers signal a more competitive league. As such, there may be a desire to see a continuation of the downward trend in championship winning percentages observed since 1977 (onset of Free Agency).

However, if, as the psychological experiments reviewed earlier suggest [3], all the golden ratio really represents is the level or threshold at which people perceive a clear imbalance, then it is this imbalance that has prompted changes (rule, equipment, strategy, materials) that strengthen weak teams and weaken strong ones. These efforts have evolved, over time, to keep the success of winning at about this level—a level at which there is a clearly superior team, but which does not make the competition seem lopsided or unfair or too discouraging, especially for fans of weaker teams. Indeed, even the winning percentage in the Post-Steroid era, all eras in fact, while they might be lower (or above) the golden ratio, they remain remarkably close to 0.618. The variability around the golden ratio that was observed by era is nothing more than the variability that is produced by the outcomes of experimentation to maintain this balance. Thus, it is possible that championship winning percentages will “naturally” reconfigure around the golden ratio, as administrators periodically tinker with rules, equipment, or policies. The test for this will come from future results. If we are correct, we should see a rise in winning percentage in the “Post Post-Steroid” era, moving into a high sport revenue era. More time (and future data) are required to confirm, and future studies should build on our analysis by considering how qualitative and quantitative measures of fan satisfaction correspond with winning percentages.

From a marketing perspective, if the golden ratio truly represents an underlying universal aesthetic [1.4], then teams should also consider other ways to enhance fan experience by capitalizing on its appeal. For example, one of the most common manifestations of the golden ratio is in architecture. Designing stadium features using the golden ratio could enhance the experience for fans who attend games in person. Logo designs and how they are positioned on uniforms are another example. Using the golden ratio to guide design and placement could also increase the aesthetic image of players and the team brand. Understanding the appeal of the ratio should prompt broader considerations as to its strategic application in marketing the product.

In sum, despite the counterintuitive idea that baseball may be attractive due to its aesthetic appeal, it seems to follow similar ratio as various form of arts. After all, as argued by John Dewey, “Acts that were primitively spontaneous are converted into means that make human intercourse more rich and gracious—just as a painter converts pigment into means of expressing an imaginative experience. Dance and sport are activities in which acts once performed spontaneously in separation are assembled and converted from raw, crude material into works of expressive art.” [27, p. 66].

## Data Availability

Publicly available datasets were analyzed in this study. This data can be found here: https://www.baseball-reference.com/.
